# Fear of Pain—and Dental Neglect

**Published:** 1917-08

**Authors:** Bernard J. Cigrand


### FEAR OF PAIN—AND DENTAL NEGLECT

BY BERNARD J. CIGRAND, M.S., D.D.S.

For scores of years we have been taught that the proper way to put metallic fillings into teeth is to so form the cavity that the metal somewhat anchored by the dove-tail principle it is held firmly in place. I fully realize what a dangerous thing it is to attempt to speak or write on cavity preparation, a subject supposed to have been reduced to an exact science, and it would seem folly to suggest any change in either technic or principle. But I am sufficiently well acquainted with the history of mankind to know that often that which seems absolutely reduced to an exact science later proves to be only a mere step in the right direction, not the goal or the final word.

Now I will not venture to alter your method of cavity formation, but I will state that a modification of the underlying principles will be a great benefit to relieve the suffering of our patrons, who are subjected, I think, to many unnecessary tortures, and in consequence remain away from the dental office, preferring ill health to cruel inflictions at our hands. I well know that many dentists resent such a charge, but the facts are, they have not received the honest confession of patrons and the public, or they would heed what I have written. And do not forget that your patients do not always make their complaints to you, but that they reserve that right to tell it to some other dentist.

While it might seem difficult to make it clear just how, when and where these modified changes could best be made, and that to hope to make the suggestions without diagrams, drawings or specimens, yet I will make it so easy that the reading of the "Evening Story" could not be more engaging—provided you are interested in the story, "How to Inflict Less Pain While Filling Teeth."

Please do not get it into your mind that all fillings must be inserted according to any set, rigid rules—notwithstanding that your teachers and famed authors have given the edict for scientific work. Sometimes artistic work outlives scientific work, and you will comprehend what I say if you read on about ten sentences further. For example, a boy came to me last fall who had, because of injuries at football, sustained a severe shock, and he was ill and debilitated and in below-par condition generally. He had been suffering some with a cavity in the mesial side of the left central; the pulp was still covered with sturdy tooth structure, and cold or hot water flushings rendered but slight pain. But he was intensely fearful of pain and the slightest jar on the tooth caused pain. Having in his earlier years been severely treated at a dental office, he was dreadfully reluctant about entering the dental chair, but finally I said to him: "I will not use the dental engine, and since you are so afraid of the drill we will not use it."

Now, scientific dentistry, so-called, of properly shaping the cavity, was entirely out of order. I decided that artistic dentistry—or medicinal dentistry—was the best, and the results were so satisfactory to both that I trust others, too, may see the good of casting aside the iron-clad rules and substitute mere easy-going regulations instead.

I did not distress him with the use of the unwelcome rubber dam, as his critical nervous condition impelled kindness in every particular. I placed a small piece of bibulous paper—thanks to Dr. E. J. Berry for that suggestion—under his upper lip, dried out the saucer-shaped cavity and with small spoons carefully removed the superficial and imperfect tissue structure, and quickly inserted—without any attempt at cavity preparation, a combination cement filling. The entire operation, including final trimming, did not exceed a half hour.

When the lad was dismissed from the chair he was astonished at the painless, simple and satisfactory process.

Now, the facts are that it was proper that I should discard all dogmatic rules, and artfully do two things—first, stop the toothache, and second, fill the tooth painlessly, and both things were easily and quickly performed.

Just before he departed I told him that when he felt stronger and so inclined, he should call and have such filling placed in the tooth as we might agree or decide to be better, and his prompt reply, "I will be glad to come back and do what you suggest," afforded me considerable comfort.

In this case I was simply artfully arranging to later put in the scientifically prepared cavity and its scientifically placed filling.

Some may say that the permanent filling should have been located at once, but I am inclined to object to that over-used term, "permanent filling," since the very name leads people to believe that it will last forever, that they will carry that filling you called permanent to the throne of God, or to the fires of Hades, where it will even resist the fusing flames.

We have by this word caused our profession untold labors and anxieties. People forget that all things which are used must materially suffer wear or that some repair is indicated where service is exacted, hence they cheerfully buy new shoes because they use them; they gladly purchase new clothes because they wear on them, and they buy all other things pertaining to the comforts of the body and expect to replace them occasionally with new ones; but not so with a *permanent filling*. That, by the very term given by us, means to endure in its present form without change, to last and continue to remain with not the slightest alteration; to exist and endlessly continue to exist. No wonder when a filling of alloy kept its place forty years and it came out the patient returned to Dr. Nels Nelson with the original dental chart marked ''one permanent amalgam filling'' and demanded him to replace it without charges, and the doctor told me: ''By George, I was compelled to put it in as I had personally condemned myself and any court would have sustained him; but believe me, I stopped marking anything—absolutely anything —operative or prosthetic, as permanent.''

Some lesson in this, and the additional lesson is that sometimes a so-called temporary filling would outlast the so-called permanent filling—depending entirely on choice of material to fit into given oral circumstances as well the manner of its technical adaptability and method of insertion.

This will strike some as a radical deviation from accepted ideals and adopted practice, but do not forget that what is accepted considered ''the thing'' today, is oft called a ''thing of the past'' tomorrow, and the only possible way for you to be near up to date is to be close to the right and the philosophical way, rather than with the popular or accepted way. Think out some of these things for yourself. Confer with your patients; they too can give you a world of information which will be of service to you. You will get from them thousands of points which you can not get in a dental textbook, since the latter only roughly, superficially and oft inaccurately attempts to demonstrate. The real truth, the actual facts and the unfailing test tube of practice will always remain—for the dentist—the mouth and its possessor, and again what often appears to us to be temporary very often proves to be enduring success, and *vice versa*. And do not forget that a cavity refilled with a good cement is better than a poorly placed gold filling. If I were to have my personal choice as to having a good cement used every two years to preserve a tooth in which the pulp was but covered with thin structure or have a metal filling placed which would not only cause pain on drinking or eating hot or cold things, I would take the cement, and though it needed to be replaced a dozen times in twenty years. As time goes on we are going to get farther away from that old bunk, permanent fillings. A guttapercha filling can be considered a permanent filling providing the patient dies the day after it is inserted; a cement filling can be pronounced a permanent filling in a soldier's mouth provided he is killed before its margins are eroded or washed away. A gold inlay can be called a permanent filling provided it does not drop out before you get through polishing it, and a gold leaf filling, too, may justly be called a permanent filling if it stays in until the patient dies. So, the long and short of the situation is that whether a filling is temporary or permanent depends on how long the patient lives rather than in the material used or the character of its insertion. But up, over and above all, get away from using the term "permanent filling." It is not only a misnomer, but gets you and your fellow practitioners into rounds of trouble all the days of the year. Call your fillings what they are intended for and do not lead the patient to believe that we do any work which can possibly rival the Creator. Do not assume to be more than the Divine. If his beautifully-formed teeth and splendidly-formed eyes and handsomely-shaped insteps give way—why may not some of our splendid products suffer a like career? Just do the best, the very best, you can, and you will be satisfied in both head and heart, and more neither king nor peasant can claim.—*Facts.*

